# Genetic tools for the development of recombinant lactic acid bacteria

**DOI:** 10.1186/s12934-021-01607-1

**Published:** 2021-06-19

**Authors:** Jiapeng Wu, Yongping Xin, Jian Kong, Tingting Guo

**Affiliations:** State Key Laboratory of Microbial Technology, Shandong University, Qingdao, 266237 People’s Republic of China

**Keywords:** Lactic acid bacteria, Food-grade microorganisms, Genome-editing tools, Probiotic characteristics, Therapeutic functionalities

## Abstract

Lactic acid bacteria (LAB) are a phylogenetically diverse group with the ability to convert soluble carbohydrates into lactic acid. Many LAB have a long history of safe use in fermented foods and are recognized as food-grade microorganisms. LAB are also natural inhabitants of the human intestinal tract and have beneficial effects on health. Considering these properties, LAB have potential applications as biotherapeutic vehicles to delivery cytokines, antigens and other medicinal molecules. In this review, we summarize the development of, and advances in, genome manipulation techniques for engineering LAB and the expected future development of such genetic tools. These methods are crucial for us to maximize the value of LAB. We also discuss applications of the genome-editing tools in enhancing probiotic characteristics and therapeutic functionalities of LAB.

## Background

Lactic acid bacteria (LAB) were unknowingly used as starters in fermented dairy products for thousands of years [1]. Toward the end of the nineteenth century, Pasteur identified lactic acid fermentation of yogurt, after which Lister obtained the first pure LAB culture [2]. Since then, LAB have been studied in depth, and their physiology and genetic characteristics were gradually revealed. Nowadays, strains of LAB play indispensable roles in the dairy industry and in the manufacture of beverages, meat and vegetables, in the production of safe, healthy, tasty, and nutritious fermented foods [3]. At the same time, LAB are normal inhabitants of the human intestinal tract and the most common microbes used as probiotics [4, 5].

With the evolution of sequencing technologies, the human gut microbiome is being decoded. Its relationships to human health have opened up the use of next-generation probiotics as therapeutics and diagnostics. In this field, strains must be safe and survive in the intestinal tract [6]. Considering these factors, LAB may serve as ideal chassis to produce functional compounds in vivo or in vitro. Moreover, LAB can directly contact with intestinal mucosa, making them the preferred carriers of antigens and medical molecules to promote mucosal immunity [7]. A full set of technologies is required to manipulate LAB for therapeutic and diagnostic purposes, including but not limited to introducing DNA into LAB cells, and site-specific chromosomal mutations, deletions, stable integrations, and insertions. In the past 30 years, the genetic toolbox for LAB has been improved by the development of various components, such as the Cre-*loxP* and λ-Red or RecET systems. Encouragingly, the clustered regularly interspaced short palindromic repeats–CRISPR-associated proteins (CRISPR–Cas) system has opened a new chapter of genome editing research. In this article, we review the technologies for manipulation of LAB genomes and their use to channel LAB as microbial cell factories and delivery vehicles for the treatment of various conditions.

## Uptake of foreign DNA by LAB

Introduction of foreign DNA into LAB is an indispensable step in genetic manipulation. Natural transformation (NT) is one of the commonest pathways that mediate horizontal gene transfer in microbial species. During NT, competence for DNA transformation is induced in response to signaling peptides referred to as competence pheromones. Competent cells generally interact with double-stranded DNA (dsDNA) in the environment, but only a single strand of this DNA is translocated into the cytoplasm. Upon uptake into the cytoplasm, this single-stranded DNA (ssDNA) is rapidly bound by proteins including the recombination protein RecA and the DNA processing protein DprA, then the ssDNA can be directly integrated into the genome. NT has been adopted as a highly efficient tool for genetic manipulation of *Streptococcus pneumoniae* for > 60 years. The master competence regulator ComX, responsible for DNA binding, uptake and recombination, has been identified in all streptococcal species [8, 9]. Therefore, NT was explored for genetic manipulation of *Streptococcus* *thermophilus* [10]. This method allows transformation of *S*. *thermophilus* with classical vectors and with linear fragments that can be directly integrated into the chromosome if they are flanked by 1-kb homologous fragments corresponding to the upstream and downstream regions where the foreign DNA needs to be inserted [10]. Moreover, the use of linear fragments allows insertion of large DNA fragments (up to 15 kb), which is challenging using plasmid vectors [11]. For some strains of *S*. *thermophilus*, the transformation rate was only 1%, thus the process could be improved [12]. In addition to streptococci, *Lactococcus lactis* can also be endowed with the ability for NT by overproduction of the master competence regulator ComX [13]. Culture conditions and regulatory mechanisms should be further explored to achieve NT in other species of LAB.

Electroporation is the simplest artificial transformation technology. Electroporation protocols have been well developed for frequently-used host LAB species, such as *Lc*. *lactis*, *Lactobacillus* *casei*, *Lb*. *plantarum*, *Lb*. *brevis* and *S*. *thermophilus*. First, cells are made competent by cultivation in medium containing hypertonic and cell wall weakening solutions, to attenuate the natural barrier to foreign DNA. Then, the competent cells are subjected to high-voltage pulses, and transient membrane pores are formed that allow the entrance of the negatively-charged DNA molecules [14, 15]. Electroporation is highly efficient. However, the electroporation protocols might not work in uncommonly-used genetic hosts such as wild-type strains isolated from the human gastrointestinal tract. Therefore, alternative natural methods, such as transduction and conjugation, are gaining renewed attention to achieve plasmid or chromosomal DNA transfer between LAB strains; the former is a phage-mediated DNA transfer method, and the latter can achieve DNA translocation between two cells that form a mating pair using two types of mobile genetic elements: conjugative plasmids and integrating conjugative elements (ICEs) [16–18]*.*

## Gene knock-out and knock-in technologies in LAB

Chromosomal disruption and integration strategies were established in LAB based on traditional non-replicative plasmids (pWV01, pG + host) and insertion sequence (IS) transposons [19–21]. To accelerate screening of recombinants, researchers introduced appropriate counterselectable markers, such as uracil-phosphoribosyltransferase (UPRT), the orotate transporter (OroP), and phenylalanyl-tRNA synthetase (PheS) [22–25]. Emerging technologies are being developed in LAB, such as dsDNA or ssDNA recombineering and CRISPR–Cas systems, which greatly simplify the procedures of gene knock-out or knock-in in LAB chromosomes and improve the efficiency of generation of target mutants.

### Recombineering

Recombineering refers to homologous recombination between exogenous DNA and the bacterial genome, to achieve gene deletion, insertion, or replacement. Recombineering includes dsDNA recombineering and ssDNA recombineering [26, 27]. DsDNA recombineering is mediated by bacteriophage-encoded recombinase systems, λ-Redαβγ and RecET. Redα/RecE is a 5′-3dsDNA exonuclease that digests exogenous dsDNA to generate 3′-ended ssDNA overhangs. Redβ/RecT is a single strand annealing protein that binds to the ssDNA overhangs and promotes strand exchange and strand invasion. Redγ inhibits the RecBCD nuclease from attacking linear DNA [28]. Thus, when dsDNA substrates are electroporated into cells, Redα/RecE digests the dsDNA to produce ssDNA overhangs, Redγ inhibits the activity of the endogenous bacterial nuclease RecBCD, and Redβ/RecT binds to the ssDNA overhangs to promote the renaturation of complementary strands and mediates DNA strand annealing and exchange reactions [28].

**Fig. 1:**
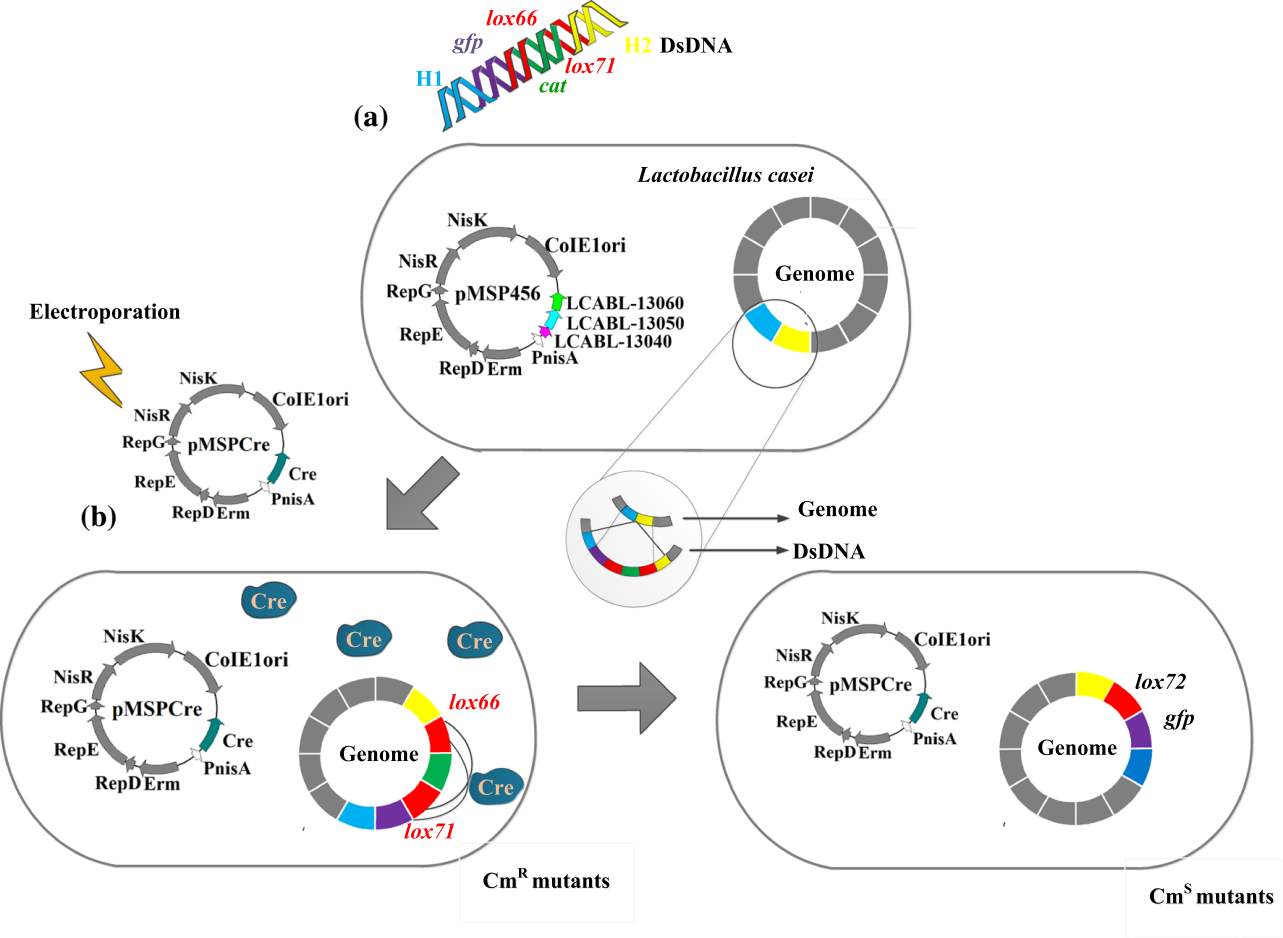
Schematic representation of dsDNA recombineering in *Lactobacillus casei*. **a** A piece of dsDNA substrate harboring the *lox66*-cat-*lox71* cassette (*lox66* and *lox71* sites, red; *cat*/Cm marker, green), *gfp* (the gene of the green fluorescence protein, purple), and DNA overhangs homologous to the genomic insertion site (H1, blue; H2, yellow) was electroporated into *Lb*. *casei* expressing an λ Red-like recombinase operon LCABL_13040-50-60. **b** Once the dsDNA substrate was integrated into the genome, the recombinant cell was endowed with chloramphenicol resistance (Cm^r^). The recombinant cell was transformed with a plasmid pMSPCre carrying the site specific recombinase Cre to direct the recombination between the *lox66* and *lox71* sites for the deletion of the Cm marker. The resultant mutant cells (Cm^s^) contained the *gfp* gene and a *lox72* site at the target site

The feasibility of λ-Red- and RecET-like systems in LAB was first reported when Yang et al. identified a λ-Red like recombinase system from a prophage of *Lb*. *plantarum* WCFS1; Lp_6040, Lp_6041, and Lp_6042 were the analogs to Redγ, Redα, and Redβ, respectively. Combining the Lp*_*6040-41-42 system with the Cre-*loxP* system (a site-specific recombinase system consisting of 34 bases) yielded efficient deletion of the glucosamine-6-phosphate isomerase gene (*gnp*) and replacement of the d-lactate dehydrogenase gene (*ldhD*) with mutation efficiencies of 95% and 75%, respectively in *Lb*. *plantarum* WCFS1 [29]. Xin et al. explored another λ-Red like recombinase system, LCABL*_*13040*-*50-60, to mediate markerless deletion of a 167-bp *galk* fragment and insertion of the green fluorescent protein gene (*gfp*) with mutation efficiency of 100% in *Lb*. *casei* BL23 (Fig. 1) [30]. Xin et al. also extended the LCABL_13040-50-60 recombination system into 12 strains of lactobacilli and one of *Lactococcus*, which broadened the host range of the recombinase system [30]. DsDNA recombineering can be used for deletions, insertions or replacements of large gene fragments (up to 4.7 kb in *Lb*. *plantarum*) by integrating dsDNAs into the genome [29]. However, the selection of positive mutants is marker-dependent, and a scar (a *loxP* site) is left at the modification locus after excision of the selection marker. ssDNA recombineering neatly avoids this problem. SsDNA recombineering only requires the overexpression of Redβ/RecT, and ssDNA can be guided by Redβ/RecT to homologous sequences on the bacterial chromosome [31]. The first successful attempt of ssDNA recombineering in LAB species was carried out in *Lb. reuteri* ATCC PTA 6475*.* Transformation of 100 μg ssDNA into *Lb*. *reuteri* after expression of RecT from *Enterococcus faecalis* (*E*. *faecalis*) yielded precise mutation with efficiency 0.4–19% [31]. Because ssDNA recombineering did not use antibiotic selection markers in *Lb*. *reuteri*, the efficiency of obtaining positive mutants was relatively low. Therefore, it is necessary to develop new screening strategies to capture positive mutants more easily.

### CRISPR–Cas-based systems

CRISPR–Cas system constitute adaptive immune systems in bacteria and archaea that can actively reject the invasion of foreign genetic elements such as phages and plasmids [32]. Since 2013, genome editing using CRISPR–Cas systems has undergone explosive growth. In particular, the type II CRISPR–Cas9 system from *S*. *pyogenes* has been exploited as a facile and programmable platform for genome editing in a sequence-specific manner in some eukaryotes and prokaryotes. The system consists of Cas9 (an endonuclease), a *trans*-activating CRISPR RNA (tracrRNA) and a precursor crRNA array containing nuclease guide sequences (spacer) interspaced by identical direct repeats [33]. The precursor crRNA is processed within repeat sequences to generate mature crRNA, which further forms a duplex with the tracrRNA. The duplex interacts with Cas9, searches present DNA for a trinucleotide protospacer adjacent motif (PAM), and binds to proximal chromosomal complementary sequences (protospacer), inducing double-stranded breaks (DSBs) in the chromosome [34]. The lethal (unless repaired) DSBs stimulate the non-homologous end joining recombination (NHEJ) (existed in rare bacteria) or homologous recombination (HR) pathway to repair the DNA lesion, and thus desired mutations can be produced [34]. Moreover, CRISPR–Cas9 can be used as a counterselectable marker, as Cas9-induced DSBs in the wild-type allele allow rapid screening of expected mutants.

**Fig. 2:**
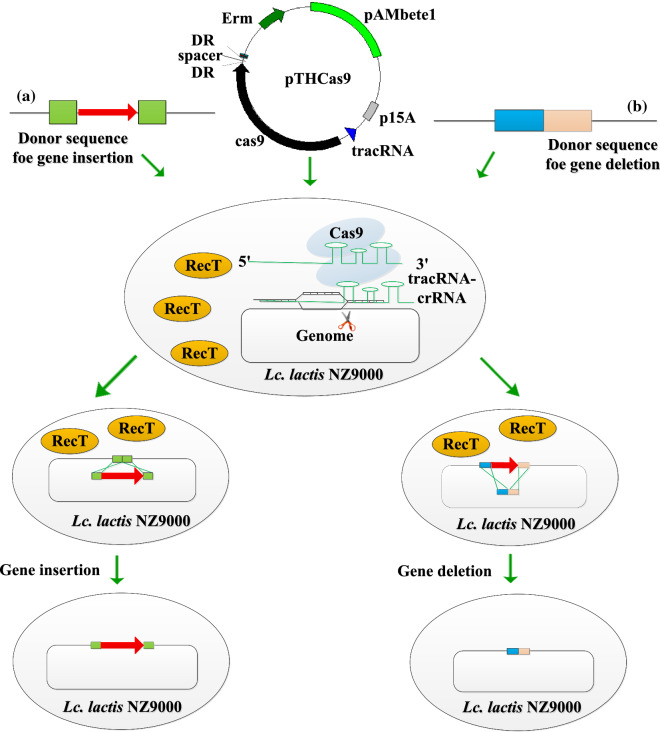
Use of CRISPR–Cas9 systems for genome editing. **a** Schematic strategies of gene insertion using RecT-assisted CRISRP–Cas9 system. **b** Schematic strategies of gene deletion using RecT-assisted CRISRP–Cas9 system. Plasmid pTHCas9 harboring the Cas9 nuclease, tracrRNA and crRNA and the donor ssDNA substrates were electroporated into *Lc*. *lactis* cells expressing RecT. After ssDNA recombineering, the positive mutants were counter selected by CRISPR–Cas9. *DR* direct repeats

In 2014, a RecT-assisted CRISPR–Cas9 approach was developed to perform codon saturation mutagenesis and gene deletions in the chromosome of *Lb*. *reuteri* ATCC PTA 6475 [35]. A similar approach was used in *Lc*. *lactis* NZ9000; seamless genomic DNA insertion or deletion (Fig. 2a, b) was efficiently accomplished within 72 h [36]*.* The CRISPR–Cas9 system was even used to modify the genome of the *Lc*. *lactis* virulent phage P2 and precise mutations were successfully achieved without the assistance of heterologous recombinases [37]. Leenay et al. used two plasmids carrying the recombineering template and CRISPR–Cas9 elements to achieve genome editing in *Lb*. *plantarum* WJL, but this failed in *Lb*. *plantarum* NIZO2877 and *Lb*. *plantarum* WCFS1, indicating that the genetic engineering feasibility of the method varied depending on the targeted gene(s) and strain [38]. Variants of Cas9 have also been developed, such as Cas9 nickase (Cas9^D10A^), which generates chromosomal single-strand breaks (nicks), circumventing the high lethality of DSBs induced by Cas9 [34]. A CRISPR–Cas9^D10A^-based plasmid was constructed for genome engineering of *Lb*. *casei* LC2W, which allowed enhanced green fluorescent protein (eGFP) gene insertion and putative uracil phosphoribosyltransferase (UPRT) gene deletions with efficiencies of 35% and 65%, respectively [39].

In addition to introducing point mutations, deletions and insertions in targeted genes, the CRISPR system can be used to regulate gene expression through CRISPR interference (CRISPRi) with catalytically inactive variants of Cas9 (dCas9), in which the endonucleolytic activity of Cas9 has been eliminated but the targeted binding function was still remains [40]. CRISPRi systems served as robust tools for transcriptional regulation of the essential cell cycle genes in *Lb*. *plantarum* [41, 42]. This work provided an ideal example of how to quickly screen both essential and nonessential genes by CRISPRi-mediated knockdowns. The CRISPRi system was also used to perform single gene or multiple genes silencing in *Lc*. *lactis* [41, 43]. Table 1 summarizes the current tools available for genome editing of LAB.

**Table 1:** Current tools available for lactic acid bacteria genome editing

Tools	Examples of partial applications	Characteristics	References
Plasmids-based allelic exchange	*Lc. lactis, S. thermophilus, E*. *faecalis*	Homologous recombination-dependent; marker free; time-consuming	[19, 20]
DsDNA recombineering	*Lb*. *plantarum*, *Lb*. *casei*	Recombinase-mediated; high efficiencies for both deletion and insertion; marker-dependent	[29, 30]
SsDNA recombineering	*Lb*. *reuteri*, *Lc. lactis*, *Lb*. *plantarum*, *Lb*. *gasseri*	Mutation efficiency 0.4–19%; applicable to genomic mutagenesis; marker free	[31]
CRISPR–Cas-assisted recombineering	*Lb*. *reuteri*, *Lc. Lactis*	High efficiency (up to 100%) for small deletions (< 1.0 kb in *Lb*. *reuteri*, < 100 bp in *Lc. lactis*); marker free	[35, 36]
CRISPR–Cas9D10A	*Lb*. *casei*	Used for both gene deletion and insertion (25–65%); simplified editing procedure; marker free	[39]
CRISPRi	*Lb*. *plantarum*, *Lc*. *lactis*	Used to repress multiple target genes simultaneously; reversible effects; precise targeting; marker free	[40–43]

### Gene integration into the chromosome

**Fig. 3:**
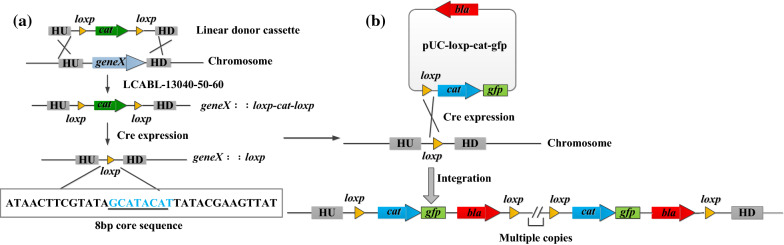
Repetitive integration system of *Lb*. *casei* BL23. **a** Prophage recombinase LCABL_13040-50-60 mediated homologous recombination, resulting in the replacement of *geneX* with the *cat* marker, while site-specific recombinase Cre subsequently eliminated the marker and a *loxP* site was left. The inserted *loxP* site was verified by sequencing analysis. **b** The *gfp* gene was repetitively integrated into the chromosome between the *loxP* sites of the integration construct pUC-lox-cat-gfp and chromosome. 40-50-60, recombinase LCABL_13040-50–60; HU and HD, up and down homologous arms; *cat*, *bla*, indicate chloramphenicol and ampicillin resistance genes respectively

LAB have relatively simple metabolic pathways and can survive in the intestinal tract. Therefore, they are ideal candidates for delivery of cytokines, antigens, and other pharmaceutical molecules [44–46]. Previously, expression of the target genes using plasmids was a common strategy for producing desired metabolites, but antibiotics must be added as selective pressure to maintain the presence of plasmids in LAB. Integration of target genes or gene clusters into the chromosome of LAB is preferable, to avoid the potential product safety risks and environmental pollution associated with antibiotic use. Using dsDNA or ssDNA recombineering strategy, genes of interest can be knocked-in to the target locus in the chromosome, but both the size and copy number of inserted genes are limited. To achieve integration of large DNA fragments or gene clusters at one or several chromosomal loci, the site-specific recombination systems can be adopted. The site-specific recombinase catalyzes the recombination between the recombinase recognition sites on a circular DNA and the chromosome. Moreover, after one round of integration, an additional recognition site is generated in the chromosome, so it is possible to achieve repetitive integration of target genes into the target sites. As proof of this concept, we constructed the recombinant *Lb*. *casei* BL23 strains in which the *gfp* gene or the fimbrial adhesin gene *faeG* from *Escherichia coli* was repetitively integrated into the chromosome using the Cre-*loxP* system. GFP and FaeG were stably expressed in the recombinant strains without supplementation of the culture with antibiotics, and the protein production was comparable to that of a plasmid-engineered strain (Fig. 3) [47].

In addition to the Cre-*loxP* system, other site-specific recombinases from LAB prophages have been developed as genomic integration tools. Researchers constructed an integration vector with several new genetic traits using both the integrase and *attP* sequences of phage ΦAT3. The integration vector was capable of stably integrating the *gfp* gene into the chromosome of lactobacilli [48]. Although the efficiencies and accuracy of site-specific recombination systems are satisfactory, their application is restricted because most LAB strains lack the necessary recognition sites in the chromosome. Fortunately, insertion of the recombinase recognition sites (*loxP*) into the chromosome provided a convenient method to integrate the targeted genes, as in reports for *Lb*. *casei* and *Lc*. *lactis* [31, 36].

## Applications of genome-editing tools in enhancing probiotic characteristics and therapeutic functionalities of LAB

The sections above describe emerging and potential tools that allow rapid, efficient genetic engineering of LAB. They enable production of tailored probiotics with specific traits, enhancing the probiotic characteristics and therapeutic functionalities of the bacteria.

### Enhanced probiotic strains and starter cultures

Bacteria must endure a variety of harsh conditions either in industrial environments or in the gastrointestinal tract, including oxidative and osmotic stress, acid and bile, pathogens, and the host immune response. These stresses pose a challenge to survival and effective colonization. Exopolysaccharides (EPS) is important for stress resistance [49]. Researchers have demonstrated that spontaneous mutations of the hypothetical membrane-anchored protein Balat_1410 and the putative tyrosine kinase EpsC altered EPS properties in *Bifidobacterium animalis* subsp. *lactis* and *Lb*. *johnsonii*, respectively, resulting in cells that were resistant to gastrointestinal stress [49, 50]. *S*. *thermophilus* cannot grow on galactose and ferments only the glucose portion of lactose; the residual galactose is excreted into the medium, which would have adverse effects on galactosemia patients. Spontaneous mutation in the *galKTEM* promoter of *S*. *thermophilus* produced a mutant strain with galactose-consuming ability [51]. Using the emerging genome editing technologies, such as the CRISPR–Cas systems, the introduction of single nucleotide mutations would undoubtedly faster than spontaneous mutation through consecutive cultures. Other galactose transformation pathways could also be introduced into *S*. *thermophilus* using genomic integration strategies [52].

### Engineered LAB for delivery of biotherapeutics

LAB are appealing as vaccine carriers as they are able to induce both mucosal and systemic immune responses, and are free from the risks of conventional attenuated live pathogens [7]. Steidler et al. reported the application of engineered *Lc*. *lactis* to secrete interleukin-10 (IL-10) for the treatment of inflammatory bowel disease (IBD) in colitis-induced mice [44]. In *Lc*. *lactis*, the essential *thyA* gene (encoding thymidylate synthase) was replaced by the IL-10-encoding gene; when deprived of thymidine or thymine, the viability of the strain decreased by several orders of magnitude, essentially preventing its accumulation in the environment [53]. *Lc*. *lactis* without *thyA* has been evaluated in human clinical trials, and even though the trial did not satisfy expectations regarding efficacy, the bio-containment strategy was highly successful [54]. Since the use of IL-10 for IBD treatment, many other cytokines have been produced in *Lc*. *lactis*, including IL-12 and IL-6 [55, 56]. Apart from delivering cytokines, LAB have also been developed as cell factories for production and delivery of allergens. For example, *Lc*. *lactis* CHW9 was used to produce peanut allergen Ara 2; *Lc*. *lactis* NZ9800 was used to deliver the major birch allergen Bet-v1; and *Lb*. *plantarum* NCL21 was used to produce a major Japanese cedar pollen allergen, Cry j1, that can suppress allergen-specific immunoglobulin E response and nasal symptoms in a murine model of cedar pollinosis [57–59].

Recombinant LAB are regarded as a potential alternatives to current therapies for type I diabetes; for example, recombinant *Lc*. *lactis* NZ9000 expressed fusion protein HSP65-6P277 to improve glucose tolerance in a mouse model [60]. In the field of anticancer therapeutics, recombinant *Lc*. *lactis* NZ9000 secreting tumor metastasis-inhibiting peptide Kisspeptin was used to inhibit HT-29 cell proliferation and migration through the induction of apoptosis pathways and by downregulating matrix metallopeptidase-9 expression [61]. Other cancer antigens expressed using *Lc*. *lactis* include an E7 antigen against human papilloma virus type-16 and a glycosylated tyrosinase related protein-2 tumor antigen against melanoma (the latter has not gone to animal trials) [62, 63]. In addition to protein and peptide-based therapeutics, metabolites with medicinal applications are produced by LAB, such as γ-amino butyric acid and hyaluronic acid. The former is a non-proteinaceous amino acid with hypotensive, anticancer, antianxiety, and diuretic properties, and the latter is a carbohydrate polymer used in wound healing and to treat dermatitis [64, 65]. The use of modified LAB as transmitters of medical molecules is very promising. Table 2 summarizes therapeutics produced by various recombinant LAB.

**Table 2:** Therapeutics produced from various recombinant lactic acid bacteria

Therapeutic products	Disorder/disease	Strains	References
Interleukin-10 (IL-10)	Inflammatory bowel disease (IBD)	*Lc*. *lactis N/S*	[44]
Interleukin-12 (IL-12)	Asthma	*Lc*. *lactis* NZ9000	[55]
Interleukin-6 (IL-6)	Adjuvant	*Lc*. *lactis* IL1403	[56]
Peanut allergen Ara2	Hypersensitivity type I	*Lc*. *lactis* CHW9	[57]
Birch allergen Betv1	Hypersensitivity type I	*Lc*.*lactis* NZ9800	[58]
Japanese cedar pollen allergen Cry j1	Hypersensitivity type I	*Lb*. *plantarum* NCL21	[59]
HSP65-6P277	Diabetes mellitus type I	*Lc*. *lactis* NZ9000	[60]
Kisspeptin	Colorectal cancer	*Lc*. *lactis* NZ9000	[61]
HPV-16-E7	HPV-16 induced cancers	*Lc*. *lactis* NZ9000	[62]
Glycosylated tyrosinase related protein-2	Skin cancer	*Lc*. *lactis* MG1363	[63]
γ-Amino butyric acid	Hypertension, anxiety	*Lb*. *pentosus* SS6	[64]
Hyaluronic acid	Wound healing, dermatitis	*Lb*. *acidophilus* PTCC1643	[65]

*N/S* not specified

## Conclusions and future perspectives

Rapid progress has been made in genetic engineering of LAB using recombineering and CRISPR-based systems. We can obtain desired mutant strains in several days, which accelerates fundamental research and functional exploitation. CRISPR-based editing tools will be further improved in LAB, including multilocus editing and Cas9-NHEJ repair, which have been achieved in *E*. *coli* [66] and *Mycobacterium tuberculosis* [67, 68]. We can also learn from new achievements with CRISPR–Cas9-based systems in eukaryotes. For example, new genetic information could be written into specified DNA sites using a dCas9 fused to an engineered reverse transcriptase in human cells [69]. Newly-reported insertion systems might also provide inspiration. CRISPR-associated transposases (CAST) have been explored in *Vibrio cholerae* and cyanobacteria. CAST were able to integrate foreign gene fragments directly into chromosomal target sites with frequencies of up to 80% without positive selection [70, 71]. The CAST loci are approximately 20 kb long and contain a Tn7-like transposase, cargo genes and V-U CRISPR system. On the basis of these characteristics, approximately 30% type V-U putative Cas protein have been identified in 171 *Lactobacillus* species [72], suggesting the presence of genetic elements for the development of non-redundant and targeted genome integration systems in LAB.

In the future, the LAB genome editing platforms will be more complete. The cloning and identification of new recombinase and CRISPR systems will undoubtedly make the genetic modification of LAB faster and easier. Integration of CRISPR-based editing with synthetic biology approaches holds promise for the development of intelligent therapeutic delivery that responds to changes in the intestinal environment, such as using pH-dependent promoters, xylose-induced expression systems, and heat shock-responsive promoters [73–75]. Although there is a long way to go before we can efficiently and reliably engineer non-model gut microorganisms, the potential benefits are considerable, and would open up new avenues for the genesis of engineered probiotic strains to improve human health with unprecedented speed, ease, and scale.

## Data Availability

Not applicable.

## Abbreviations

LAB: Lactic acid bacteria
NT: Natural transformation
ICEs: Integrating conjugative elements
IS: Insertion sequence
UPRT: Uracil-phosphoribosyltransferase
OroP: Orotate transporter
PheS: Phenylalanyl-tRNA synthetase
CRISPR–Cas: The clustered regularly interspaced short palindromic repeats–CRISPR-associated proteins
tracrRNA: *trans*-Activating CRISPR RNA
PAM: Protospacer adjacent motif
DSBs: Double-stranded breaks
NHEJ: Non-homologous end joining recombination
HR: Homologous recombination
eGFP: Enhanced green fluorescent protein
CRISPRi: CRISPR interference
EPS: Exopolysaccharides
IL-10: Interleukin-10
IBD: Inflammatory bowel disease
IL-12: Interleukin-12
IL-6: Interleukin-6
CAST: CRISPR-associated transposases
